# MRSA-ST398 in livestock farmers and neighbouring residents in a rural area in Germany

**DOI:** 10.1186/1753-6561-5-S6-P169

**Published:** 2011-06-29

**Authors:** B Bisdorff, J Scholhölter, K Claußen, M Pulz, D Nowak, K Radon

**Affiliations:** 1 Institute for Environmental&Occupational Medicine, Universirt Hospital of Munich, LMU, Munich, Germany; 2 Governmental Institute of Public Health of Lower Saxony, Hannover, Germany

## Introduction / objectives

The aim of the study was to establish, for the first time the prevalence and risk factors associated with MRSA-ST398 carriage, a strain usually found in animals, in a rural population with occupational livestock contact as well as neighbouring residents.

## Methods

A cross-sectional survey was out in a pig and poultry dense area in Germany. 2756 questionnaires and self-sampling nasal swabs were sent out in the winter of 2009/2010.

## Results

Overall 1872 out of 2753 (response 70%) people, aged between 26 and 53 years of age, took part in the study. Overall, 1.5% of the tested population without and 24% with occupational livestock contact tested positive for MRSA and MRSA-ST398 for the former; MRSA-ST398 only for the latter. The group without occupational livestock contact were 3.8 times (95% CI 1.5-9.3) more likely to be colonized if a household member had livestock contact; 3.2 times (95% CI 1.4-7.4) more likely if they regularly carried out private farm visits (e.g. to buy eggs or milk). In the group with occupational livestock contact, pig contact had an Odds Ratio of 7.1 (95% CI 2.9-17.2) for MRSA-ST398 acquisition.

## Conclusion

This is the first study establishing a MRSA prevalence of 1.5% within the general population without occupational livestock contact. The study furthermore confirmed already established risk factors for those with and those without occupational livestock contact. It also suggested private farms visits as new potential risk factor for MRSA colonization for the group without occupational contact. More research however into establishing the exact transmission routes and foremost into measures to prevent the spread of the bacterium in the farming environment is still required.

## Disclosure of interest

None declared.

